# *trans*-Bis(1,1,1,5,5,5-hexa­fluoro­pentane-2,4-dionato-κ^2^ *O*,*O*′)bis­(4-methyl-1,2,3-selenadiazole-κ*N* ^3^)copper(II)

**DOI:** 10.1107/S1600536810001297

**Published:** 2010-01-30

**Authors:** Neil M. Boag, Andrew D. Jackson, Paul D. Lickiss, Richard D. Pilkington, Alan D. Redhouse

**Affiliations:** a School of Computing, Science and Engineering, University of Salford, Salford M5 4WT, England; b Department of Chemistry, Imperial College London, South Kensington, London SW7 2AZ, England

## Abstract

In the title compound, [Cu(C_5_HF_6_O_2_)_2_(C_3_H_4_N_2_Se)_2_], the Cu^II^ atom (site symmetry 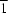) is coordinated by two *O*,*O*′-bidentate 1,1,1,5,5,5-hexa­fluoro-2,4-penta­nedione (hp) ligands and two 4-methyl-1,2,3-selenadiazole mol­ecules, resulting in a slightly distorted *trans*-CuN_2_O_4_ octa­hedral geometry in which the *cis* angles deviate by less than 3° from 90°. The selenadiazole plane is canted at 73.13 (17)° to the square plane defined by the penta­nedionate O atoms. The F atoms of one of the hp ligands are disordered over two sets of sites in a 0.66 (3):0.34 (3) ratio. There are no significant inter­molecular inter­actions in the crystal.

## Related literature

Similar stuctures are exhibited by bis­(hexa­fluoro­penta­dionato) copper complexes of imidazole (Colacio *et al.,* 2000 ▶), pyrazole (Kogane *et al.,* 1990 ▶; Fokin *et al.*, 2002 ▶) and substituted pyridines (De Panthou *et al.,* 1996 ▶; Iwahori *et al.,* 2001 ▶; Sano *et al.*, 1997 ▶).
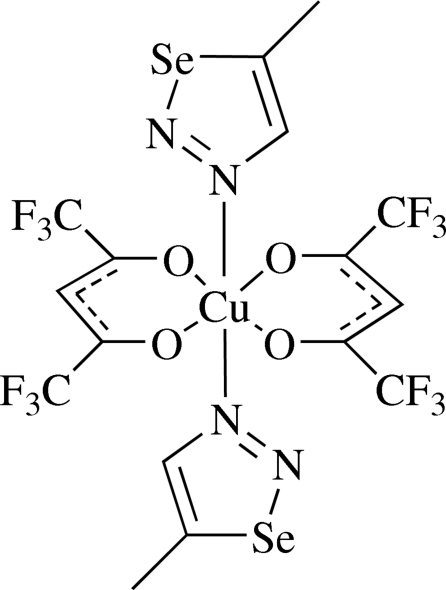


## Experimental

### 

#### Crystal data

[Cu(C_5_HF_6_O_2_)_2_(C_3_H_4_N_2_Se)_2_]
*M* *_r_* = 771.74
Monoclinic, 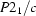

*a* = 8.191 (2) Å
*b* = 14.390 (4) Å
*c* = 11.429 (4) Å
β = 104.86 (3)°
*V* = 1302.1 (7) Å^3^
*Z* = 2
Mo *K*α radiation
μ = 3.75 mm^−1^
*T* = 293 K
0.25 × 0.25 × 0.25 mm

#### Data collection

Nicolet R3m/V diffractometer
Absorption correction: ψ scan (*SHELXTL*; Sheldrick, 2008 ▶) *T*_min_ = 0.755, *T*_max_ = 0.793
3218 measured reflections
3014 independent reflections
1894 reflections with *I* > 2σ(*I*)
*R*_int_ = 0.048
3 standard reflections every 97 reflections  intensity decay: none

#### Refinement

*R*[*F* ^2^ > 2σ(*F* ^2^)] = 0.055
*wR*(*F* ^2^) = 0.126
*S* = 1.03
3014 reflections
215 parameters
H atoms treated by a mixture of independent and constrained refinement
Δρ_max_ = 0.44 e Å^−3^
Δρ_min_ = −0.42 e Å^−3^

### 

Data collection: *XSCANS* (Siemens, 1996 ▶); cell refinement: *XSCANS*; data reduction: *XSCANS*; program(s) used to solve structure: *SHELXL97* (Sheldrick, 2008 ▶); program(s) used to refine structure: *SHELXL97* (Sheldrick, 2008 ▶); molecular graphics: *SHELXTL* (Sheldrick, 2008 ▶); software used to prepare material for publication: *SHELXL97* (Sheldrick, 2008 ▶).

## Supplementary Material

Crystal structure: contains datablocks global, I. DOI: 10.1107/S1600536810001297/hb5239sup1.cif

Structure factors: contains datablocks I. DOI: 10.1107/S1600536810001297/hb5239Isup2.hkl

Additional supplementary materials:  crystallographic information; 3D view; checkCIF report

## Figures and Tables

**Table 1:** Selected bond lengths (Å)

Cu—O1	1.967 (3)
Cu—O2	1.981 (3)
Cu—N1	2.391 (4)

**Fig. 1.**
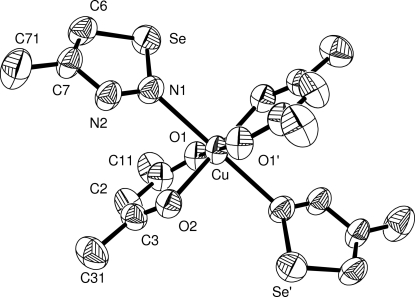
The molecular structure of (I), with 50% probability displacement ellipsoids. Hydrogen atoms and the fluorine atoms have been excluded for clarity.

## supplementary crystallographic information

### Comment

The title molecule, [Cu(C_3_HF_6_O_2_)_2_(C_3_H_4_N_2_Se)_2_], (I) was prepared as a potential precursor to CuInSe_2_. The molecule (Fig. 1) is centrosymmetric resulting in pairs of equivalent ligands lying *trans* to each other in a slightly distorted octahedral coordination geometry in which the *cis* angles deviate less than 3° from right angles. The Cu is bound to N1 of the selenadiazole whose plane is canted at 73.13 (0.17) ° to the square plane defined by the pentanedionato oxygen atoms. The Cu—N bond is elongated. There are no intermolecular interactions.

### Experimental

A solution of 4-methyl-1,2,3-selenadiazole (4.65 g, 0.032 mol) in dichloromethane was added dropwise to a solution of Cu(hfac)_2_.xH_2_O (7.84 g, 0.016 mol) in dichloromethane/toluene (50 ml) at 273 K in the absence of light. The mixture was stirred for 12 h, the solvent removed *in vacuo* and the residue dissolved in warm toluene. Slow cooling afforded olive-green parallelepipeds of (I). Yield 8.13 g (65%), mpt. 354–356 K. Found: C, 24.86; H, 1.24; N, 7.31%. Calc.for C_16_H_12_CuF_12_N_4_O_4_Se_2_: C, 24.90; H, 1.31; N, 7.26%. µ 2.15 BM.

### Refinement

All H atoms were placed in calculated positions and refined using a riding model. All other hydrogen atoms were located and fully refined.

### Crystal data

**Table:**

[Cu(C_5_HF_6_O_2_)_2_(C_3_H_4_N_2_Se)_2_]	*D*_x_ = 1.968 Mg m^−^^3^
*M**_r_* = 771.74	Melting point: 355 K
Monoclinic, *P*2_1_/*c*	Mo *K*α radiation, λ = 0.71073 Å
*a* = 8.191 (2) Å	Cell parameters from 20 reflections
*b* = 14.390 (4) Å	θ = 7.9–14.5°
*c* = 11.429 (4) Å	µ = 3.75 mm^−^^1^
β = 104.86 (3)°	*T* = 293 K
*V* = 1302.1 (7) Å^3^	Parallelpiped, green
*Z* = 2	0.25 × 0.25 × 0.25 mm
*F*(000) = 742	

### Data collection

**Table:**

Nicolet R3m/V diffractometer	*R*_int_ = 0.048
graphite	θ_max_ = 27.6°, θ_min_ = 2.3°
profile data from θ/2θ scans	*h* = −2→10
Absorption correction: ψ scan (*SHELXTL*; Sheldrick, 2008)	*k* = −6→18
*T*_min_ = 0.755, *T*_max_ = 0.793	*l* = −14→14
3218 measured reflections	3 standard reflections every 97 reflections
3014 independent reflections	intensity decay: none
1894 reflections with *I* > 2σ(*I*)	

### Refinement

**Table:**

Refinement on *F*^2^	Primary atom site location: structure-invariant direct methods
Least-squares matrix: full	Secondary atom site location: difference Fourier map
*R*[*F*^2^ > 2σ(*F*^2^)] = 0.055	Hydrogen site location: inferred from neighbouring sites
*wR*(*F*^2^) = 0.126	H atoms treated by a mixture of independent and constrained refinement
*S* = 1.03	*w* = 1/[σ^2^(*F*_o_^2^) + (0.0459*P*)^2^ + 1.964*P*] where *P* = (*F*_o_^2^ + 2*F*_c_^2^)/3
3014 reflections	(Δ/σ)_max_ < 0.001
215 parameters	Δρ_max_ = 0.44 e Å^−^^3^
0 restraints	Δρ_min_ = −0.42 e Å^−^^3^

### Special details

**Table:**

Geometry. All e.s.d.'s (except the e.s.d. in the dihedral angle between two l.s. planes) are estimated using the full covariance matrix. The cell e.s.d.'s are taken into account individually in the estimation of e.s.d.'s in distances, angles and torsion angles; correlations between e.s.d.'s in cell parameters are only used when they are defined by crystal symmetry. An approximate (isotropic) treatment of cell e.s.d.'s is used for estimating e.s.d.'s involving l.s. planes.

### Fractional atomic coordinates and isotropic or equivalent isotropic displacement parameters (Å^2^)

**Table:**

	*x*	*y*	*z*	*U*_iso_*/*U*_eq_	Occ. (<1)
Cu	0.0000	0.0000	0.5000	0.0467 (2)	
Se	0.11516 (8)	0.14840 (4)	0.26686 (5)	0.0667 (2)	
N1	0.0700 (6)	0.1433 (3)	0.4188 (4)	0.0564 (11)	
N2	0.0391 (6)	0.2234 (3)	0.4541 (4)	0.0572 (11)	
C6	0.0853 (8)	0.2732 (4)	0.2739 (5)	0.0628 (15)	
C7	0.0470 (7)	0.2966 (4)	0.3777 (5)	0.0577 (13)	
C71	0.0087 (11)	0.3911 (4)	0.4164 (6)	0.093 (2)	
H71A	−0.0143	0.4322	0.3479	0.140*	
H71B	0.1040	0.4139	0.4772	0.140*	
H71C	−0.0881	0.3882	0.4489	0.140*	
O1	−0.1707 (5)	−0.0178 (2)	0.3457 (3)	0.0546 (9)	
O2	−0.1614 (5)	0.0692 (2)	0.5702 (3)	0.0520 (8)	
C1	−0.3071 (7)	0.0274 (4)	0.3148 (5)	0.0582 (14)	
C11	−0.4037 (9)	0.0109 (5)	0.1836 (6)	0.0771 (19)	
F11	−0.5552 (6)	0.0472 (5)	0.1545 (4)	0.157 (3)	
F12	−0.3218 (6)	0.0490 (3)	0.1084 (3)	0.1142 (15)	
F13	−0.4170 (6)	−0.0780 (3)	0.1545 (4)	0.1076 (14)	
C2	−0.3728 (8)	0.0879 (4)	0.3859 (5)	0.0674 (16)	
C3	−0.2960 (7)	0.1034 (4)	0.5075 (5)	0.0541 (13)	
C31	−0.3885 (10)	0.1722 (5)	0.5750 (7)	0.0782 (19)	
F311	−0.318 (2)	0.2515 (8)	0.581 (3)	0.145 (9)	0.66 (3)
F312	−0.5418 (12)	0.1700 (18)	0.5425 (19)	0.161 (10)	0.66 (3)
F313	−0.349 (2)	0.1452 (18)	0.6957 (10)	0.137 (6)	0.66 (3)
F321	−0.300 (2)	0.210 (2)	0.663 (3)	0.105 (10)	0.34 (3)
F322	−0.520 (6)	0.141 (2)	0.591 (5)	0.18 (2)	0.34 (3)
F323	−0.457 (6)	0.2452 (19)	0.4941 (19)	0.138 (14)	0.34 (3)
H2	−0.475 (7)	0.118 (4)	0.347 (5)	0.065 (17)*	
H6	0.085 (9)	0.314 (5)	0.207 (7)	0.12 (3)*	

### Atomic displacement parameters (Å^2^)

**Table:**

	*U*^11^	*U*^22^	*U*^33^	*U*^12^	*U*^13^	*U*^23^
Cu	0.0603 (5)	0.0401 (4)	0.0375 (4)	0.0048 (4)	0.0085 (4)	−0.0043 (4)
Se	0.0834 (5)	0.0652 (4)	0.0568 (4)	0.0044 (3)	0.0276 (3)	−0.0066 (3)
N1	0.072 (3)	0.051 (3)	0.051 (2)	0.001 (2)	0.023 (2)	0.004 (2)
N2	0.081 (3)	0.047 (3)	0.047 (2)	0.002 (2)	0.023 (2)	0.005 (2)
C6	0.080 (4)	0.063 (4)	0.049 (3)	−0.001 (3)	0.022 (3)	0.012 (3)
C7	0.080 (4)	0.047 (3)	0.046 (3)	0.002 (3)	0.014 (3)	0.009 (2)
C71	0.152 (7)	0.059 (4)	0.076 (4)	0.002 (4)	0.044 (5)	0.001 (3)
O1	0.065 (2)	0.051 (2)	0.0436 (18)	0.0042 (18)	0.0060 (17)	−0.0060 (16)
O2	0.064 (2)	0.047 (2)	0.0444 (18)	0.0014 (17)	0.0132 (17)	−0.0044 (15)
C1	0.067 (4)	0.058 (3)	0.045 (3)	0.003 (3)	0.007 (3)	−0.002 (2)
C11	0.077 (4)	0.092 (5)	0.052 (4)	0.012 (4)	−0.003 (3)	−0.016 (3)
F11	0.098 (3)	0.260 (7)	0.081 (3)	0.080 (4)	−0.033 (2)	−0.062 (4)
F12	0.149 (4)	0.133 (4)	0.052 (2)	−0.001 (3)	0.011 (2)	0.013 (2)
F13	0.130 (4)	0.102 (3)	0.072 (2)	−0.014 (3)	−0.008 (2)	−0.024 (2)
C2	0.061 (4)	0.074 (4)	0.060 (3)	0.013 (3)	0.003 (3)	−0.014 (3)
C3	0.056 (3)	0.048 (3)	0.061 (3)	−0.001 (3)	0.019 (3)	−0.010 (3)
C31	0.081 (5)	0.070 (5)	0.089 (5)	−0.002 (4)	0.032 (4)	−0.030 (4)
F311	0.164 (16)	0.064 (6)	0.23 (2)	0.004 (7)	0.095 (18)	−0.043 (9)
F312	0.045 (6)	0.27 (2)	0.158 (13)	0.041 (8)	−0.001 (6)	−0.123 (14)
F313	0.147 (12)	0.188 (15)	0.088 (6)	0.053 (10)	0.052 (7)	−0.030 (8)
F321	0.067 (11)	0.12 (2)	0.104 (17)	0.027 (14)	−0.015 (11)	−0.071 (14)
F322	0.23 (4)	0.132 (17)	0.27 (5)	−0.10 (2)	0.22 (4)	−0.10 (2)
F323	0.21 (3)	0.101 (14)	0.094 (12)	0.105 (18)	0.034 (16)	0.019 (10)

### Geometric parameters (Å, °)

**Table:**

Cu—O1	1.967 (3)	C1—C2	1.390 (8)
Cu—O2	1.981 (3)	C1—C11	1.524 (8)
Cu—N1	2.391 (4)	C11—F11	1.308 (7)
Se—C6	1.817 (6)	C11—F13	1.318 (8)
Se—N1	1.867 (4)	C11—F12	1.336 (8)
N1—N2	1.268 (6)	C2—C3	1.389 (8)
N2—C7	1.380 (6)	C2—H2	0.95 (6)
C6—C7	1.345 (7)	C3—C31	1.565 (8)
C6—H6	0.97 (8)	C31—F312	1.214 (12)
C7—C71	1.488 (8)	C31—F311	1.273 (12)
C71—H71A	0.96	C31—F313	1.389 (15)
C71—H71B	0.96	C31—F321	1.204 (17)
C71—H71C	0.96	C31—F322	1.23 (2)
O1—C1	1.262 (6)	C31—F323	1.418 (17)
O2—C3	1.252 (6)		
			
O1—Cu—O2^i^	88.06 (14)	O1—C1—C11	113.2 (5)
O1—Cu—O2	91.94 (14)	C2—C1—C11	119.5 (5)
O1—Cu—N1	87.15 (15)	F11—C11—F13	108.2 (6)
O1^i^—Cu—N1	92.85 (15)	F11—C11—F12	105.9 (6)
O2^i^—Cu—N1	91.44 (15)	F13—C11—F12	105.0 (6)
O2—Cu—N1	88.57 (15)	F11—C11—C1	114.0 (5)
C6—Se—N1	86.4 (2)	F13—C11—C1	112.8 (6)
N2—N1—Se	111.3 (3)	F12—C11—C1	110.4 (6)
N2—N1—Cu	125.0 (3)	C3—C2—C1	122.8 (6)
Se—N1—Cu	121.3 (2)	C3—C2—H2	121 (3)
N1—N2—C7	116.6 (4)	C1—C2—H2	116 (3)
C7—C6—Se	110.6 (4)	O2—C3—C2	128.1 (5)
C7—C6—H6	126 (4)	O2—C3—C31	115.7 (5)
Se—C6—H6	123 (4)	C2—C3—C31	116.3 (6)
C6—C7—N2	115.2 (5)	F312—C31—F311	117.2 (13)
C6—C7—C71	127.2 (5)	F312—C31—F313	104.9 (12)
N2—C7—C71	117.5 (5)	F311—C31—F313	102.0 (11)
C7—C71—H71A	109.5	F312—C31—C3	115.2 (8)
C7—C71—H71B	109.5	F311—C31—C3	109.0 (8)
H71A—C71—H71B	109.5	F313—C31—C3	107.1 (8)
C7—C71—H71C	109.5	F321—C31—F322	114 (2)
H71A—C71—H71C	109.5	F321—C31—F323	105.4 (16)
H71B—C71—H71C	109.5	F322—C31—F323	99 (2)
C1—O1—Cu	123.9 (3)	F321—C31—C3	115.3 (10)
C3—O2—Cu	123.2 (3)	F322—C31—C3	113.4 (14)
O1—C1—C2	127.4 (5)	F323—C31—C3	107.9 (9)
			
C6—Se—N1—N2	0.8 (4)	Cu—O1—C1—C11	171.1 (4)
C6—Se—N1—Cu	164.0 (3)	O1—C1—C11—F11	171.4 (6)
O1—Cu—N1—N2	109.9 (4)	C2—C1—C11—F11	−8.8 (10)
O1^i^—Cu—N1—N2	−70.1 (4)	O1—C1—C11—F13	47.5 (8)
O2^i^—Cu—N1—N2	−162.1 (4)	C2—C1—C11—F13	−132.7 (6)
O2—Cu—N1—N2	17.9 (4)	O1—C1—C11—F12	−69.6 (7)
O1—Cu—N1—Se	−50.9 (3)	C2—C1—C11—F12	110.2 (7)
O1^i^—Cu—N1—Se	129.1 (3)	O1—C1—C2—C3	−3.2 (11)
O2^i^—Cu—N1—Se	37.1 (3)	C11—C1—C2—C3	177.1 (6)
O2—Cu—N1—Se	−142.9 (3)	Cu—O2—C3—C2	11.9 (8)
Se—N1—N2—C7	−1.0 (6)	Cu—O2—C3—C31	−168.1 (4)
Cu—N1—N2—C7	−163.4 (4)	C1—C2—C3—O2	1.3 (10)
N1—Se—C6—C7	−0.5 (5)	C1—C2—C3—C31	−178.8 (6)
Se—C6—C7—N2	0.1 (7)	O2—C3—C31—F312	−145.3 (18)
Se—C6—C7—C71	−178.2 (6)	C2—C3—C31—F312	34.8 (19)
N1—N2—C7—C6	0.6 (8)	O2—C3—C31—F311	80.6 (17)
N1—N2—C7—C71	179.1 (6)	C2—C3—C31—F311	−99.4 (16)
O2^i^—Cu—O1—C1	−164.5 (4)	O2—C3—C31—F313	−29.0 (14)
O2—Cu—O1—C1	15.5 (4)	C2—C3—C31—F313	151.0 (13)
N1^i^—Cu—O1—C1	107.0 (4)	O2—C3—C31—F321	24 (3)
N1—Cu—O1—C1	−73.0 (4)	C2—C3—C31—F321	−156 (3)
O1—Cu—O2—C3	−16.9 (4)	O2—C3—C31—F322	−110 (3)
O1^i^—Cu—O2—C3	163.1 (4)	C2—C3—C31—F322	71 (3)
N1^i^—Cu—O2—C3	−109.8 (4)	O2—C3—C31—F323	142 (2)
N1—Cu—O2—C3	70.2 (4)	C2—C3—C31—F323	−38 (3)
Cu—O1—C1—C2	−8.6 (8)		

Symmetry codes: (i) −*x*, −*y*, −*z*+1.
